# A Novel Sprouted Oat Fermented Beverage: Evaluation of Safety and Health Benefits for Celiac Individuals

**DOI:** 10.3390/nu13082522

**Published:** 2021-07-23

**Authors:** Natalia Aparicio-García, Cristina Martínez-Villaluenga, Juana Frias, Laura Crespo Perez, Cristina Fernández Fernández, Claudio Alba, Juan Miguel Rodríguez, Elena Peñas

**Affiliations:** 1 Institute of Food Science, Technology and Nutrition (ICTAN-CSIC), Juan de la Cierva 3, 28006 Madrid, Spain; n.aparicio@ictan.csic.es (N.A.-G.); c.m.villaluenga@csic.es (C.M.-V.); frias@ictan.csic.es (J.F.); 2 Department of Gastroenterology, University Hospital Ramón y Cajal, Ctra. De Colmenar Viejo M-607, 28034 Madrid, Spain; lcreper@yahoo.es (L.C.P.); crisf99@hotmail.com (C.F.F.); 3 Department of Nutrition and Food Science, Complutense University of Madrid, 28040 Madrid, Spain; claudioalbarubio@gmail.com (C.A.); jmrodrig@ucm.es (J.M.R.)

**Keywords:** oat, germination, fermentation, beverage, celiac disease, gluten-free diet, microbiota

## Abstract

The safety and health effects for celiac people of a novel beverage (SOFB) developed from sprouted oat flour by fermentation with *Lactobacillus plantarum* was explored. In vitro reactivity against anti-gliadin antibodies (AGA) and antioxidant/anti-inflammatory potential of SOFB in RAW 264.7 macrophages and Caco-2 cells were evaluated. Immunoreactivity against AGA and antioxidant activity were not detected in SOFB, but it exhibited significant anti-inflammatory activity. The tolerability and impact of SOFB consumption for 6 months on nutritional status and intestinal microbiota composition were investigated in 10 celiac adults (five treated and five control). SOFB consumption did not adversely affect duodenal mucosa nor the total IgA or anti-tissue transglutaminase antibody (IgA-tTG) levels in celiac participants, but it significantly decreased total cholesterol levels at all sampling times and folic acid levels at the end of the study compared to the placebo beverage. SOFB administration also shifted gut microbiota, leading to a higher relative abundance of some beneficial bacteria including the genera *Subdoligranulum*, *Ruminococcus* and *Lactobacillus* in the SOFB group. This study provides supporting evidence of the safety of health benefits of a novel functional beverage produced from sprouted oat.

## 1. Introduction

Celiac disease (CD) is a chronic immune-mediated disorder resulting from gluten exposure in genetically predisposed individuals carrying the human leukocyte antigen (HLA)-DQ2 and HLA-DQ8 haplotypes. Dietary gluten induces an autoimmune-like reaction in CD individuals that results in mucosal inflammation, small intestinal villous atrophy, and crypt hyperplasia, causing impaired nutrient absorption and a wide spectrum of clinical manifestations [1,2]. Tissue transglutaminase (tTG) has been shown to exert two crucial roles in CD: as a deamidating enzyme of proline-rich gluten peptides, thus enhancing any gluten immunostimulatory effects, and as an auto-antigen [3]. The current gold standard for CD diagnosis is represented by the combination of mucosal histological changes detected by duodenal biopsy and measurement of concentration of IgA-tTG antibodies [4].

The mainstay of CD treatment is a lifelong avoidance of gluten-containing cereals. Long-term adherence to a strict gluten-free (GF) diet causes clinical, serological, and histological remission in CD patients [1]. GF food consumption by non-CD populations has burgeoned in recent years due to the belief that they are healthier than their gluten-containing counterparts and can contribute to weight loss [5]. The increased interest on GF foods by both CD and non-CD consumers has resulted in a strong growth of the GF foods market. Despite tremendous popularity of processed GF foods, concern has been raised in recent years over their nutritional quality. Several studies have documented the poorer nutritional value of GF foods compared to their regular analogues, due to their lower protein, fiber and micronutrient content, and higher levels of saturated fats, salt, and sugar [6]. This fact, together with the need of excluding nutritious gluten-containing whole grains richer in fiber often, causes an imbalance of nutritional status in CD individuals [6,7].

The inclusion of oat among GF foods has been accepted by the European Commission Regulation n° 828/2014 if its gluten content does not exceed 20 mg/kg. Despite the European Regulation, this cereal has been traditionally excluded from a GF diet in Southern European countries due to the discrepant scientific findings regarding oat safety for CD people. Even though a few studies have reported harmful effects of oat in a small number of CD adults due to the induction of intraepithelial lymphocytosis and production of avenin-reactive mucosal T-cells [8,9], evidence pointing to a lack of oat toxicity in CD children and adults has also been provided in well-documented studies [10,11,12,13]. The controversial scientific results regarding oat safety and the frequent oat contamination with gluten-containing cereals, together with the existence of in vitro data indicating the existence of some immunogenic oat varieties for CD patients [14], suggest the need to evaluate the safety of novel oat varieties intended for GF food development. The inclusion of oat in GF products may offer numerous nutritional advantages due to its higher content of protein, fiber, micronutrients, and bioactive compounds than refined GF cereals [15].

Germination has emerged in recent years as a novel, sustainable, economical, and technological approach to boost the nutritional and health-promoting properties of cereals. Recent studies from our group have demonstrated that optimization of germination conditions allows to increase the levels of protein, essential amino acids, micronutrients, polyunsaturated fatty acids, gamma aminobutyric acid (GABA), and the antioxidant potential in oat [15]. In a previous publication, sprouted oat flour obtained in optimized germination conditions was used as raw material for the production of a novel GF fermented beverage (SOFB) with good physicochemical, nutritional, and sensory properties [16].

However, its potential health benefits and lack of toxicity for celiac people were not evaluated. Therefore, the aim of the present investigation was to explore the cellular antioxidant and anti-inflammatory activity of the novel SOFB developed and the in vitro immunoreactivity against AGA. Moreover, the safety of SOFB and their effects on nutritional serum biomarkers and the composition of intestinal microbiota were studied in CD adults through an intervention study.

## 2. Materials and Methods

### 2.1. Chemicals, Reagents and Standards

All chemicals used were provided by Sigma-Aldrich (Madrid, Spain) unless otherwise specified. GF sucralose (Nutrisun GmbH & Co KG, Seevetal, Germany), sodium bicarbonate (Nortem Chem S.L., Cádiz, Spain), and the GF almond powdered beverage (EcoMil, Murcia, Spain) were purchased from a local supermarket. 

### 2.2. Preparation of Sprouted Oat Flour

Dehulled GF oat grains variety Meeri were supplied by IBS Foods Solutions (Barcelona, Spain). Grains were germinated in a thermostatically-controlled germination cabinet (model G-120, Snijders Scientific, The Netherlands) at 18 °C for 4 days, as earlier reported [15]. Sprouted grains were freeze-fried, milled in an electric grinder (Taurus, Oliana, Spain), and sieved thought 0.3 mm-pore size sieve. The resultant sprouted oat flour was packed under vacuum conditions in plastic bags and stored at -20 °C until further use.

### 2.3. Bacterial Strain Activation and Inoculum Preparation

The potential probiotic strain *Lactobacillus plantarum* WCFS1 was kindly provided by Prof. M. Kleerebezem (Wageningen University & Research, The Netherlands) and it was maintained in 50% glycerol solution (*v*/*v*) at −80 °C. The inoculum was prepared in de Man, Rogosa, and Sharpe (MRS) broth (Pronadisa, Madrid, Spain), as previously described [16].

### 2.4. Manufacture of SOFB

Sprouted oat flour was thermally treated (90 °C, 30 min) to reduce the microbial load and remove potential pathogens. After cooling, sprouted oat flour was mixed with tap water (18% *w*/*v*), sucralose (0.2% *w*/*v*), and salt (0.1% *w*/*v*) in a glass jar with a screw cap, and the suspension was shaken for 2 h at 22 °C using an orbital shaker. Then, the oat suspension was heated (90 °C, 25 min), cooled to room temperature, and passed through a sterile nylon cloth (200 μm diameter, Alcavida, Barcelona, Spain). Further, sodium bicarbonate (0.35% *w*/*v*) and starter culture inoculum (0.7% *v*/*v*) were added to the formulation mixture. Fermentation was performed at 30 °C for 4 h and 140 rpm in an orbital shaker. Viable counts of *L. plantarum* WCFS1 at the end of fermentation ranged 8.7–8.9 CFU/mL, depending on the fermentation batch. SOFB was distributed in sterile twist-off cap glass bottles (200 mL/bottle) and stored at 4 °C until the intervention study. A volume of 100 mL of SOFB was freeze-dried for further analysis. Nutritional and bioactive composition of SOFB was described in a previous study [16].

### 2.5. Manufacture of Placebo Beverage for the Intervention Study

A commercial GF almond powdered beverage (EcoMil, Murcia, Spain) was used as placebo in the intervention study. The composition of the powdered drink was the following: partially defatted instant almonds (60%), corn maltodextrin, rice syrup, agave syrup, almond oil, and natural almond flavoring. For beverage preparation, the powdered beverage was mixed with boiling water (3% *w*/*v*) and shaken at 30 °C for 1 h and 140 rpm in an orbital shaker. The blend was then filtered through a sterile nylon cloth, with the same characteristics than that used for SOFB preparation. The placebo drink was distributed in sterile twist-off cap glass bottles (200 mL/bottle) and stored at 4 °C. The nutritional information (per 200 mL) of the placebo beverage, prepared as indicated, was the following: 2.9 Kcal, 1.2 g of fat, 3.6 g of carbohydrates, 0.8 g of protein, and 10.32 mg of salt.

### 2.6. Analysis of Gluten Content

The absence of gluten in raw/sprouted oat flour and SOFB was confirmed by two ELISA kits: Glutentox ELISA Competitivo (Biomedal, Seville, Spain) and INgezim Gluten Quick (Ingenasa, Madrid, Spain), as illustrated in Figure S1.

### 2.7. Evaluation of SOFB Protein Profile by Sodium Dodecyl Sulfate Polyacrylamide Gel Electrophoresis (SDS-PAGE)

The protein pattern of SOFB prolamin fraction was evaluated by SDS-PAGE under non-reducing conditions and was compared to those of raw/sprouted oat flours and wheat flour. Prolamin fraction was extracted by suspending 100 mg of oat/wheat flours or freeze-dried SOFB in 1.5 mL of 60% of ethanol (*v*/*v*). Mixtures were vortexed, sonicated for 5 min in an ultrasonic water bath (J. P. Selecta, Barcelona, Spain), and shaken (1.000 rpm, 1 h, 20 °C). After centrifugation (13,000 rpm, 5 min, 20 °C) in a Sorvall RC RC 6 Plus centrifuge (Thermo Fisher Scientific, Madrid, Spain), supernatants were evaporated under vacuum conditions (Rotavapor^®^ R-300, BÜCHI Labortechnik AG, Flawil, Switzerland). Dry extracts were dissolved in 100 μL of bidistilled water and diluted in a NuPAGE^®^ LDS sample buffer 1x (Thermo Fisher Scientific) (1:3 *v*/*v*). Prolamin extracts (15 μL/well) or Novex^®^ Sharp Prestained Protein Standard (5 μL/well) were loaded on NuPAGE^®^ Novex 4–12% Bis-Tris Gels (Thermo Fisher Scientific). Gels were placed in XCell-sure lock Mini-Cell (Thermo Fisher Scientific) and run at 200 V for 35 min, stained with SimplyBlue SafeStain (Thermo Fisher Scientific), and distained with distilled water.

### 2.8. Determination of SOFB Immunotoxicity

The immunoreactivity of raw/sprouted oat flours (ROF/SOF) and SOFB was evaluated by western blotting using a commercially available antibody developed against gluten gliadins (AGA) and wheat flour as positive control (Figure S1). After SDS-PAGE, proteins were transferred onto a PVDF membrane using a Trans-Blot Turbo Transfer System (Biorad Laboratories, Hercules, CA, USA). PVDF membranes were blocked with 5% defatted dry milk (Nestlé España, Barcelona, Spain) for 1 h and washed three times (10 min) with Tris Buffer Saline-Tween 20 (TBST). Then, membranes were incubated overnight at 4 °C with a rabbit AGA antibody labelled with horseradish peroxidase. After washing, PVDF membranes were incubated with horseradish peroxidase chemiluminescent substrate (Pearce ECL Western Blotting Substrate, Thermo Fisher Scientific) for 5 min at room temperature. Pictures of membranes were taken using a ChemDoc XRS+ Imaging System (Bio-Rad).

### 2.9. Determination of SOFB Antioxidant and Antiinflammatory Activities

In vitro antioxidant and anti-inflammatory activities were evaluated in SOFB and compared to those of two different beverages: a beverage obtained from raw (ungerminated) oat flour (ROB) and another beverage obtained from sprouted oat flour (SOB), in order to elucidate the contribution of germination and fermentation processes in SOFB bioactivity (Figure S1).

#### 2.9.1. Cell Lines

Antioxidant and anti-inflammatory activities were evaluated in RAW 264.7 murine macrophages, provided by the American Type Culture Collection (Rockville, MD, USA) and human intestinal Caco-2 cells, kindly provided by Dr. Hernández-Ledesma (CIAL-CSIC). RAW 264.7 macrophages and Caco-2 cells were routinely maintained in Dulbecco’s Modified Eagles’s Medium (DMEM, Lonza, Madrid, Spain) and Minimum Essential Medium α (Gibco, NY, USA), respectively, both supplemented with 10% fetal bovine serum (FBS, Lonza) and 1% penicillin/streptomycin (10,000 U/mL, Life Technologies, NY, USA), in a humidified incubator with a modified atmosphere (37 °C, 5% CO_2_, 95% O_2_, 90% humidity). RAW 264.7 macrophages were seeded at approximately 5 × 10^4^ cells/well into sterile 96-well plates. Cells were incubated for 24 h prior to use in the assays. Caco-2 cells were kept under sub-confluence with trypsin/EDTA (Lonza) and were allowed to differentiate to enterocytes by incubation for 10 days. The seeding density in 96-well plates was 1 x 10^5^ cells/well. Culture medium was changed every 2 days for both cell lines.

#### 2.9.2. In Vitro Gastrointestinal Digestion

ROB, SOB, and SOFB were digested following the protocol recently described by Brodkorb et al. [17] with slight modifications. Briefly, 1 g of freeze-dried beverages was dissolved in water, in order to obtain a paste-like consistency, and incubated (1:1 *v*/*v*) with a simulated salivary fluid (pH 7.0) and α-amylase (75 U/mL) for 2 min. Subsequently, oral phases were mixed (1:1 *v*/*v*) with simulated gastric fluid (pH 3.0) and pepsin (2000 U/mL), and incubated for 2 h. At this point, three gastric digests (ROBg, SOBg, and SOFBg, each one in duplicate) were inactivated, adjusting the pH to 7.0 with 1 M NaOH and immediately frozen at −80 °C. The rest of the samples were mixed (1:1 *v*/*v*) with simulated intestinal fluid (pH 7.0) and pancreatin (100 U of trypsin/mL). After 2 h of incubation, samples were frozen at −80 °C, obtaining three gastrointestinal digests (ROBi, SOBi, and SOFBi, each one in duplicate). Gastric and gastrointestinal digests were freeze-dried and kept at −20 °C under vacuum until their analysis.

#### 2.9.3. Cell Treatments

Freeze-dried gastric and gastrointestinal digests of ROB, SOB, and SOFB were dissolved in culture medium and filtered through sterile membranes (0.22 μm pore size). Digests were tested in duplicate at a concentration range from 0.5 to 10 mg/mL. Cells were treated for 24 h unless otherwise specified. The RAW 264.7 cell line was also treated with 10 μg/mL of lipopolysaccharide (LPS) from *Escherichia coli* O55:B5 in all experiments.

#### 2.9.4. Cell Viability

3-(4,5-dimethylthiazol-2-yl)-5-(3-carboxymethoxyphenyl)-2-(4-sulfophenyl)-2H-tetrazolium in the inner salt (MTS) assay was used to evaluate the cell viability of both cell lines after treatments, using cells grown only with culture medium as negative control. Positive control for RAW 264.7 macrophages consisted of non-treated cells incubated with LPS. This assay was also employed to study the cytotoxicity of *tert*-butyl hydroperoxide (*t*-BOOH) in Caco-2 cells during experiments aimed at evaluating antioxidant activity of oat-derived beverages. Previously, different concentrations of the pro-oxidant agent (0.5–5 mM) were studied on cells. After incubation (3 h), cell viability and ROS production were measured to select the *t*-BOOH concentration that resulted in a significant cytotoxic effect. To evaluate cytoprotective effects of oat beverages against oxidative stress, Caco-2 cells were treated with samples for 21 h, followed by an additional exposure with 5 mM *t*-BOOH for 3 h. In all experiments, after cell treatment, culture medium was removed, and cells were washed with PBS (Lonza, Madrid, Spain). Subsequently, 100 μL of serum-free medium and 20 μL of Cell Titer 96 AQueous^®^ One Solution were added and incubated under standard conditions for 45 min. Absorbance was measured at 515 nm in a Synergy HT microplate reader (BioTek, Winooski, VT, USA). Caco-2 cell viability was calculated considering the viability of negative control as 100%, whereas, to calculate RAW 264.7 macrophages, the viability of positive control was used. Experiments were carried out in triplicate and results were expressed as the mean of the two duplicates for each sample as % cell viability. 

#### 2.9.5. In Vitro Determination of Reactive Oxygen Species (ROS) Concentration

Determination of intracellular ROS levels was carried out using the DCFH-DA probe, which is oxidized by intracellular ROS generating the fluorescent compound DCF. To evaluate the effect of ROB, SOB, and SOFB beverages on ROS generation, supernatants of pre-treated Caco-2 cells were discarded and cells were washed with PBS. Subsequently, 200 μL of serum-free medium and 12.5 μL of 170 μM DCFH-DA were added per well and incubated under standard conditions for 30 min. Then, culture medium was removed and cells were washed again with PBS. Fluorescence was measured at 485 nm/528 nm (Biotek Synergy Microplate Reader) after adding serum-free medium (200 μL/well). For RAW 264.7 macrophages, the DCFH-DA probe was put into cells grown before treatment, as previously described. Cells washed with PBS were then treated with ROB, SOB, and SOFB digests and LPS for 24 h. Finally, fluorescence was measured, and the media were collected and stored at −80 °C for anti-inflammatory activity assay. 

Additional experiments were carried out to evaluate the cytoprotective effect of oat beverages on Caco-2 cells in stressful conditions. For that purpose, a cell model of oxidative stress induced by *t*-BOOH was used. Different concentrations of the pro-oxidant agent (0.5–5 mM) were studied on cells with the DCFH-DA probe previously incorporated. Immediately after *t*-BOOH incubation (3 h), fluorescence was measured and the *t*-BOOH concentration to use was selected. Caco-2 treatment with digest extracts was carried out for 21 h, followed by an additional 3 h with 5 mM *t*-BOOH, having incorporated the DCFH-DA probe just before oxidant treatment. Thereafter, fluorescence was measured, as mentioned above. For calculations, negative control was used in all experiments with Caco-2 cells and positive control for RAW 264.7 assay. Experiments were conducted in triplicate, expressing the results as % fluorescence units.

#### 2.9.6. Anti-Inflammatory In Vitro Assay

LPS-stimulated RAW 264.7 were used to evaluate ROB, SOB, and SOFB anti-inflammatory activity. Culture media collected after ROS measurement was employed to quantify the pro-inflammatory cytokines TNF-α and interleukin (IL)-6 by two commercial ELISA kits (Diaclone, Besançon, France) following the manufacturer instructions. Experiments were performed in duplicate and results were expressed as pg/mL.

### 2.10. In Vivo Evaluation of Safety and Health Benefits of SOFB in CD Individuals 

#### 2.10.1. Study Population and Design

Ten celiac adults (22–64 years) with a previously biopsy-proven diagnosis of CD who adhered to a strict GFD for at least 2 years were recruited at University Ramón y Cajal Hospital (Madrid, Spain) and Celiac and Gluten-Sensitive Association (Madrid, Spain). Patients who had other chronic diseases (type I diabetes, inflammatory bowel disease, or food allergies), pregnant/breastfeeding women, and patients who had consumed antibiotics and/or probiotics within the two months previous to the beginning of the study were excluded. Patients who accepted to participate were interviewed in order to collect demographic data and other information relevant for the study and they also filled structured gastrointestinal symptom (GIQLI) and celiac disease (CD-QOL) quality of life questionnaires as well as a 3-day food diary. Participants were randomly divided into two groups: treated (SOFB) and control groups. SOFB-group individuals consumed 200 mL of SOFB daily, while patients enrolled in the control group received 200 mL/day of placebo GF beverage (Figure S1). Participants consumed the beverage at the time of day that they chose alone or accompanied by other foods. The duration of the intervention was 6 months, and participants maintained a strict GF diet during the whole study period. Participants received seven bottles every week, containing SOFB or placebo beverages that were stored under refrigeration from their manufacture until their consumption. Study compliance was checked by dietary diaries and by returned empty SOFB bottles. The study design, participant recruitment, and sample collection were carried out in accordance with the Declaration of Helsinki and were approved by the Ethical Committees of CSIC and University Ramón y Cajal Hospital. A written informed consent was obtained from all enrolled participants.

Clinical and immunological markers of CD activation and nutritional/health status were monitored at 0, 2, 4, and 6 months. At every time point, body weight was recorded, blood and faecal samples were drawn for further serological and microbiological analysis, respectively, and GIQLI/CD-QOL questionnaires and a 3-day food diary were gathered. A small bowel mucosal biopsy from the second duodenal part and duodenal bulb was performed to each participant at the beginning and completion of the study.

#### 2.10.2. Serological and Histological Analysis

Blood samples were collected in EDTA tubes and were centrifuged. Plasma samples were stored at −80 °C until analysis. The levels of serum glucose, iron, ferritin, vitamin B_12_, folic acid, hemoglobin, triglycerides, and total cholesterol were quantified in blood samples from all participants. Total IgA and IgA anti-tTG antibodies were also measured by ELISA and immunofluorescence, respectively. Histological samples taken by mucosal endoscopic biopsy were analyzed by the Pathological Anatomy Service of University Ramón y Cajal Hospital. Standard microscopic examination was performed for evidence of morphological damage. Total Intraepithelial lymphocytes (IELs) and IEL subsets (TCRγδ+ and NK-like IEL) were quantified in histological samples by flow cytometry, as previously reported [18]. 

#### 2.10.3. Fecal Samples Collection and Metataxonomic Analysis

Feces from all study participants were collected in sterile containers and immediately stored at −80 °C. After thawing at room temperature, 1 g of fecal samples was used for DNA extraction following the protocol of QIAamp DNA Stool Mini Kit (QIAGEN, Hilden, Germany), as previously described [19]. DNA concentration was estimated using a Nanodrop ND-1000 UV Spectrophotometer (Nano-Drop Technologies, Wilmington, DE, USA).

The V3–V4 hypervariable region of the gene 16S rRNA was amplified according to Klindworth et al. [20] and sequenced using the Illumina MiSeq System with Illumina MiSeq pair-end protocol (Illumina Inc., San Diego, CA, USA) at the facilities of the Scientific Park of Madrid (Spain). Raw sequences were demultiplexed and quality-filtered with the Illumina MiSeq Reporter analysis software. Bioinformatic analysis of sequences were performed using QIIME 2 (v. 2019.1) pipelines. The denoising step was performed with DADA2. In order to discard nucleotides, in which median quality was Q20 or below, the reads were truncated at position 280 and 270 for the forward and the reverse reads, respectively, and their first 10 nucleotides were trimmed. Taxonomy was assigned to each amplicon sequence variants (ASVs) with the q2-feature-classifier, classify-sklearn, naive Bayes taxonomy classifier, using the SILVA 138 reference database. Subsequent bioinformatic analysis was conducted using R version 3.5.1 (R Core Team, 2013; https://www.R-project.org, accessed on 15 December 2020). A decontam package was used in order to identify, visualize, and remove contaminating DNA.

A table of ASVs, genera, and phyla count sequences per sample was generated, and bacterial taxa abundances were normalized to the total number of sequences in each sample (relative abundance). Alpha diversity was assessed using the Shannon and Simpson diversity indexes. For the beta diversity studies, a quantitative (relative abundance) and a qualitative (presence/absence) analysis for the bacterial profiles were performed with the Bray–Curtis index and binary Jaccard index, respectively. Principal coordinates analysis (PCoA) was performed in order to plot patterns of bacterial profiles through the Bray–Curtis and binary Jaccard distance matrices containing the dissimilarity value for each pairwise sample comparison.

### 2.11. Statistical Analysis

Statistica 7.0 (StatSoft Europe, Possmoorweg, Germany) was used for statistical analysis of the results. Clinical and immunological data from the intervention study were expressed as the mean ± standard deviation. Results from metataxonomic analysis were expressed as the median and interquartile range (IQR). Normal distribution of variables was checked by the Shapiro–Wilk test. Since they were not distributed normally, comparison between two groups was performed by means of non-parametric approaches (Mann–Whitney U test). The Friedman test was applied to evaluate differences during the follow-up within groups. Principal components analysis (PCoA) was performed to examine similarities in data from metataxonomic analysis between the control and SOFB groups. Plotting for microbiome results were performed in the R environment using R version 3.5.1 with library ggplot2. Differences were considered statistically significant at *p* ≤ 0.05.

## 3. Results

### 3.1. Evaluation of SOFB Gluten Content and Immunotoxicity

SOFB gluten content was assessed by two commercial ELISA kits and was found to be lower than 9 ppm (results not shown), indicating that SOFB can be considered a GF drink.

**Figure 1:**
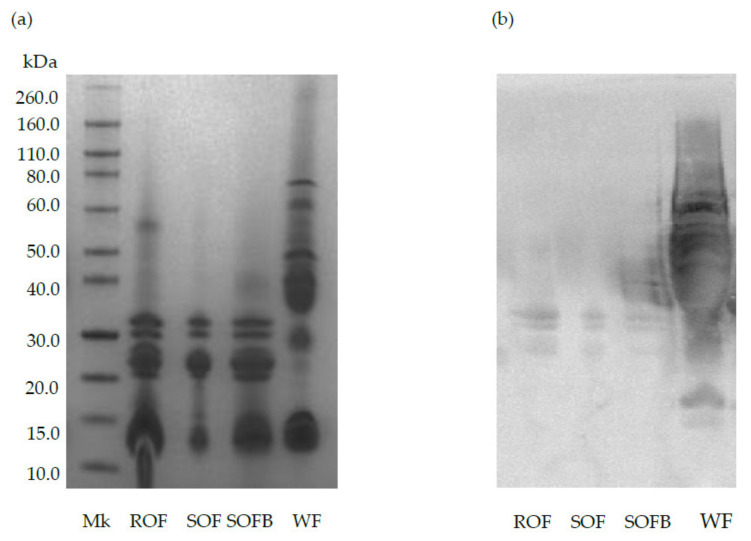
SDS-PAGE pattern (**a**) and immunochemical reactivity against AGA (**b**) of raw oat flour (ROF), sprouted oat flour (SOF), sprouted oat fermented beverage (SOFB) and wheat flour (WF, positive control). MK: molecular weight marker.

Figure 1 shows the protein profile and immunoreactivity against AGA of raw/sprouted oat flours, SOFB, and wheat (positive control). The characteristic bands corresponding to avenins can be observed in ROF, SOF, and SOFB at molecular weights ranging from 14 to 32 kDa (Figure 1a). As expected, wheat prolamins exhibited strong reactivity against AGA (lane WF), while a negligible immunochemical reaction against this polyclonal antibody was observed in ROB, SOB, and SOFB (Figure 1b).

### 3.2. SOFB Did Not Protect Caco-2 Cells against t-BOOH-Induced Oxidative Stress 

**Figure 2:**
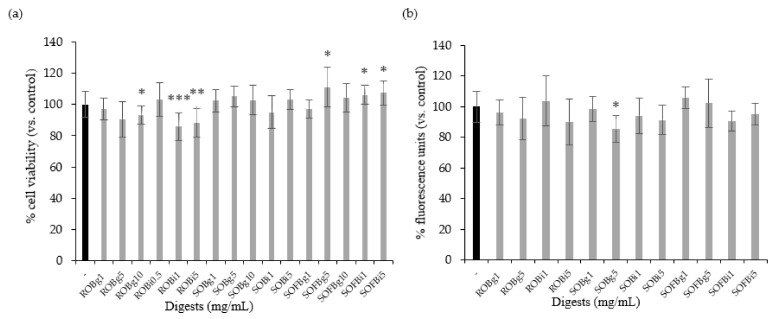
Effect of treatment with 0.5–10 mg/mL of gastric (g) and intestinal (i) digests of ROB, SOB, and SOFB for 24 h on cell viability (**a**) and intracellular ROS generation (**b**) in Caco-2 cells. Data represent the mean ± standard deviation of six biological replicates per condition. Results were expressed as percentage of cell viability (**a**) and fluorescence units (**b**) versus control cells, considered as 100%. Significant differences versus non-treated control cells are depicted as * (*p* < 0.05), ** (*p* < 0.01), and *** (*p* < 0.001). ROB: raw oat beverage; SOB: sprouted oat beverage; SOFB: sprouted oat fermented beverage.

Firstly, it was necessary to determine whether the doses of oat beverage digests used in cell experiments affected cell viability and intracellular ROS levels. Therefore, Caco-2 cells were treated with ROB, SOB, and SOFB gastric (g) and intestinal (i) digests at a concentration range of 0.5–10 mg/mL for 24 h. Oat beverage digests did not show cytotoxic effects (Figure 2a, *p* > 0.05), excepting ROB gastric and intestinal digests, which showed a lower cell viability than control cells when they were applied at concentrations ≥ 1 mg/mL. Intracellular ROS levels in Caco-2 cells treated with increasing concentrations of oat beverages did not show significant differences compared to untreated cells (*p* > 0.05, Figure 2b), with the exception of SOB gastric digest at 5 mg/mL that slightly reduced intracellular ROS (*p* < 0.05). Based on observations regarding cell viability, doses ≥ 1 mg/mL were not included in further experiments.

**Figure 3:**
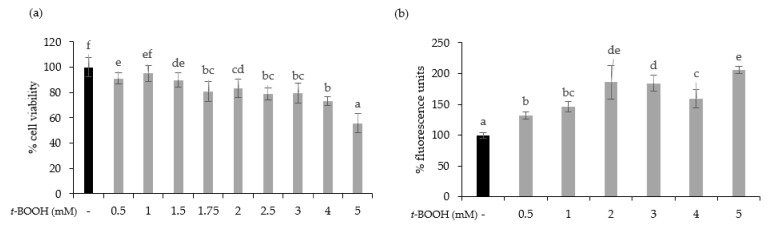
Effect of exposure of Caco-2 cells to 0.5–5 mM *tert*-butyl hydroperoxide (t-BOOH) for 3 on cell viability (**a**) and ROS generation (**b**). Data represent the mean ± standard of 4 biological replicates per condition. Different letters above bars denote statistically significant differences, *p* < 0.05.

To test the cytoprotective effect of oat beverages, a model of oxidative stress induced by *t*-BOOH was used. Different *t*-BOOH concentrations (0.5–5 mM) were tested to assure that toxicity by oxidative stress on Caco-2 cells was produced in a timeline of 3 h. Results showed that cell viability was dose-dependently reduced after exposure to *t*-BOOH, with a cytotoxic effect becoming more evident at concentrations of 4 and 5 mM, in which cell viability reached 73.04% and 55.82%, respectively (Figure 3a). This dose-dependent cytotoxic effect was consistent with an increasing trend in intracellular ROS production that reached a plateau when concentrations of *t*-BOOH were ≥2 mM (Figure 3b). Based on these results, further experiments were performed using 5 mM *t*-BOOH.

**Figure 4:**
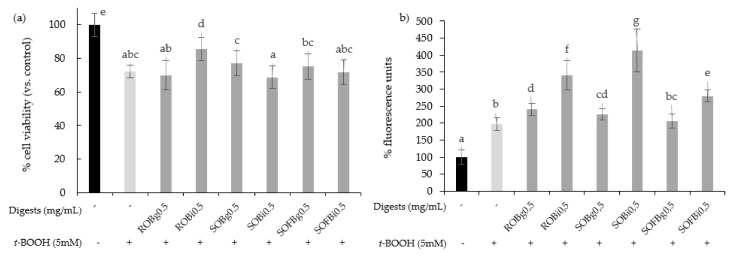
Effect of ROB, SOB, and SOFB on cell viability (**a**) and ROS generation (**b**) in *t*-BOOH exposed Caco-2 cells. Cells were pretreated with 0.5 mg/mL (g) and intestinal (i) digests of oat drinks for 21 h followed by exposure to 5 mM *t*-BOOH for 3 h. Data represent the mean ± standard deviation of six biological replicates per condition. Different letters denote statistically significant differences, *p* ≤ 0.05. ROB: raw oat beverage; SOB: sprouted oat beverage; SOFB: sprouted oat fermented beverage.

To determine whether oat beverages have a cytoprotective effect against stressful oxidant conditions, cells were treated with 0.5 mg/mL for 21 h and subsequently exposed to 5 mM *t*-BOOH for 3 h. To study the influence of the different food-processing operations (germination and fermentation) applied to obtain the final SOFB, cytoprotective effect of ROB, SOB, and SOFB was evaluated and compared. Results showed that only ROB intestinal digest significantly inhibited *t*-BOOH-induced cell death (Figure 4a), indicating that oat germination and subsequent beverage fermentation resulted in losses of ROB cytoprotective effect. As an index of the overall redox cell state, intracellular ROS generation in Caco-2 cells preincubated with oat beverages for 21 h before *t*-BOOH treatment was evaluated (Figure 4b). In general, all the digests produced an increase in ROS levels in Caco-2 cells versus control cells exposed to *t*-BOOH, with significant differences among samples. ROS accumulation became more evident when cells were exposed to intestinal digests as compared with gastric digests. Moreover, SOFB treatments were characterized by lower intracellular ROS levels compared to ROB and SOB. The increase in intracellular ROS levels was not accompanied by a decline in cell viability exhibited by oat beverages (Figure 4), although a cellular antioxidant effect could not be confirmed for any of the oat beverages tested in the present study.

### 3.3. SOFB Attenuates Inflammatory Response in LPS-Induced RAW 264.7 Macrophages

**Figure 5:**
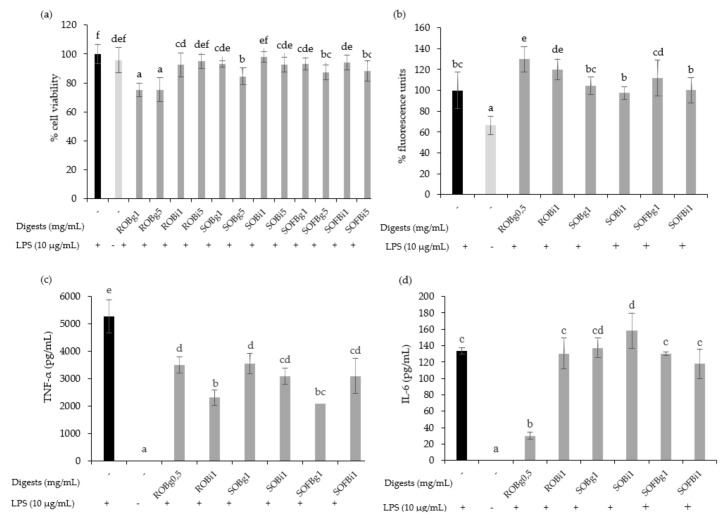
Effect of 24 h treatment of LPS (10 μg/mL) stimulated RAW 264.7 cells with 0.5–5 mg/mL of gastric (g) and intestinal (i) digest of ROB, SOB and SOFB on cell viability (**a**), intracellular ROS generation (**b**), TNF-α concentration (**c**) and IL-6 concentration (**d**). Data represent the mean ± standard deviation of 6 biological replicates per condition to (**a**,**b**) and 4 replicates to (**c**) and (**d**). Different letters denote statistically significant differences, *p* < 0.05. ROB: raw oat beverage; SOB: sprouted oat beverage; SOFB: sprouted oat fermented beverage.

The cytotoxicity of gastric and intestinal digests of oat beverages was evaluated in RAW 264.7 cells at doses ranging from 0.5 up to 5 mg/mL after 24 h post-treatments. Generally, the results showed that digests of ROB, SOB, and SOFB did not induce cytotoxic effects at doses up to 1 mg/mL, while higher concentrations (5 mg/mL) diminished cell viability for most treatments (Figure 5a). On the basis of these results, non-cytotoxic doses for both gastric and intestinal digests of each oat beverage were selected for further assays.

It is well known that macrophages are stimulated by LPS increasing intracellular ROS levels and releasing pro-inflammatory cytokines such as IL-1β, IL-6, IL-12, IL-23, and TNF-α, resulting in inflammation. To evaluate the effect of oat beverages in modulating the inflammatory response in RAW 264.7 macrophages, cells were co-treated with ROB, SOB, and SOFB, and LPS for 24 h. No differences in the intracellular ROS accumulation were observed after treatment with gastric and intestinal digests of oat beverages compared to control LPS-stimulated macrophages, with the exception of ROB digests which increased ROS levels (Figure 5b, *p* < 0.05). These findings are consistent with the experiments in Caco-2 cells confirming that oat beverages were ineffective in attenuating cellular oxidative stress. In contrast, it should be noted that TNF-α production was significantly inhibited by gastric and intestinal digests of all oat beverages tested (Figure 5c), making the anti-inflammatory activity of intestinal and gastric digests of ROB and SOFB slightly higher. This decreasing trend was also observed for the release of IL-6 only in the case of ROB gastric digest (Figure 5d).

### 3.4. In Vivo Evaluation of SOFB Safety and Health Benefits in CD Subjects

#### 3.4.1. Patient Baseline Characteristics

Study participants were celiac patients adhering to a GF diet for more than 2 years. The demographic characteristics of participants at baseline are detailed in Table 1. There were no statistically significant differences (*p* < 0.05) in gender distribution, age, duration of GF diet, weight, height, and body mass index between both groups.

#### 3.4.2. SOFB Safety Assessment in CD Subjects

The levels of total serum IgA and IgA anti-tTG antibodies in CD adult patients included in the study are shown in Table 2. Total IgA antibodies did not significantly differ (*p* ≤ 0.05) between the control and SOFB groups neither at the beginning nor after SOFB intervention. Furthermore, all subjects were negative for IgA anti-tTG at the beginning of the study (IgA anti-tTG titter < 10 U/mL), suggesting the good compliance to strict GF diet in the study participants. IgA anti-tTG antibodies remained negative in both patient groups, and no significant differences (*p* ≤ 0.05) between them were observed throughout the study period (Table 2).

All study participants exhibited a normal small bowel mucosal villous morphology at the beginning of the study as a result of a long-term GFD (>8 years as an average, Table 1). Modification of the villous architecture was not observed after the intervention period in none of the groups (results not shown), indicating that SOFB did not cause a detrimental effect in duodenal mucosa of celiac participants.

The results of IEL immunophenotype study in duodenal histological samples are summarized in Table 3. At baseline, both the control and SOFB groups exhibited a percentage of total IEL, TCR γδ+ IEL, and NK-like IEL (expressing CD103 but not CD3) in second duodenal portion and duodenal bulb ranging 8–20%, 19–28%, and 3–6%, respectively. No significant changes (*p* > 0.05) in the IEL lymphogram were observed at the end of the study period, in either of the groups.

#### 3.4.3. SOFB Effects on Body Weight and Serological Markers of Nutritional Status in CD Subjects

The effects of SOFB consumption on body weight and serum parameters associated with nutritional/health status are presented in Table 4. At baseline, no significant differences were observed between the two study groups for any of the parameters studied with the exception of triglycerides levels, which were lower (*p* ≤ 0.05) in the SOFB group.

When compared to baseline values, the control and SOFB groups did not show significant (*p* > 0.05) modification of body weight after intervention for 2, 4, and 6 months (Table 4). Furthermore, SOFB consumption did not influence the serum levels of glucose, iron, ferritin, vitamin B_12_, and hemoglobin, and no significant differences (*p* > 0.05) were found between the SOFB and control groups at the different time points of the study (Table 4). Folic acid and triglycerides levels were not significantly different (*p* > 0.05) in the SOFB group as compared with the control group at 2 and 4 months, but statistically significant (*p* ≤ 0.05) lower values in the former group were noticed for triglycerides at 0 and 6 months and for folic acid at the end of the study. Interestingly, SOFB intake resulted in significant reductions (*p* ≤ 0.05) of total serum cholesterol levels in relation to the control group at the second month of the trial and the differences were maintained until the end of the study (Table 4).

#### 3.4.4. Metataxonomic Analysis of Intestinal Microbiota in CD Subjects

Forty fecal samples corresponding to the participants of the study (10) taken at four different time points (0, 2, 4, and 6 months) were analyzed. A total of 1,706,576 high-quality filtered sequences were obtained from the 40 samples studied, and the number of sequences ranged from 36,397 to 48,201 per sample (median: 42,664; interquartile ranges (IQR): 39,786–46,134). 

**Figure 6:**
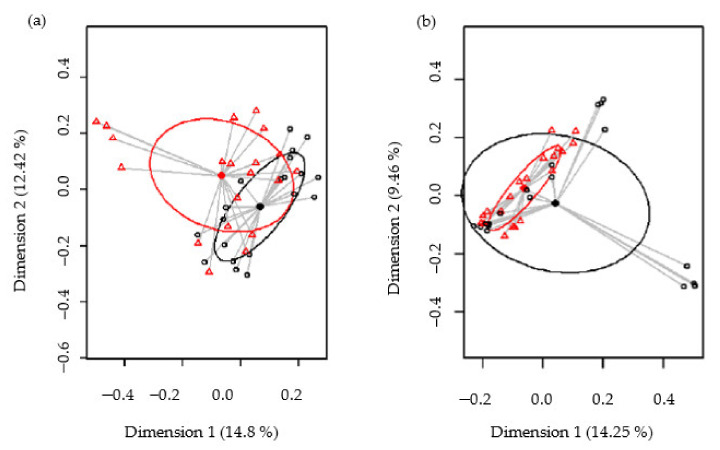
Comparison of the beta diversity for the control and SOFB groups at the ASVs level. Principal coordinate analysis (PCoA) plots of bacterial profiles based on the Bray–Curtis similarity analysis (relative abundance) (**a**) and based on the Jaccard’s coefficient for binary data (presence or absence) (**b**). The values on each axis label in graphs represent the percentage of the total variance explained by that axis. Control group (black circles); SOFB group (red triangles).

Biodiversity of the samples, as measured using the Shannon and Simpson diversity indices, was not significantly different between the control and SOFB groups (*p* = 0.74 and *p* = 0.41, respectively) (Figure S2, Supplementary Materials). The beta diversity was analyzed in order to establish differences in the microbiome profiles between both participating groups. At the ASV level, the PCoA plots of the Bray–Curtis distance matrix (relative abundance) and the presence/absence of ASVs sequences (binary Jaccard distance matrix) revealed that most of the clustered samples, according to the treatment and the differences between the two groups, were statistically significant (*p* < 0.001 and *p* < 0.001, respectively) (Figure 6).

A total of 12 phyla were identified in fecal samples. The phyla *Firmicutes* was the most abundant in study participants, followed by *Actinobacteriota*, *Bacteroidota*, *Proteobacteria*, and *Fusobacteriota*. There was a significant effect of SOFB consumption on Fusobacteriota (*p* = 0.011) (Table 5), while no statistical differences were observed for the other phyla. Similarly, the analysis of relative abundance of the 20 most abundant genera (TSS; total-sum normalization) revealed some statistical differences between the control and SOFB groups. Control patients were characterized by a lower *Subdoligranulum*, *Ruminococcus*, and *Lactobacillus* abundance (*p* = 0.002, *p* < 0.001 and *p* = 0.028, respectively) and a higher *Anaerostipes* abundance (*p* = 0.02) (Table 5). Samples of both groups also contained operational taxonomic units (OTUS) that were annotated to minor phyla having low relative abundance. Sequences belonging to unclassified genera were also observed in both sample groups (Table 5).

Due to the significant differences in bacteriome composition found between both patients’ groups, an exploratory analysis looking at the relation between gut microbiota and the treatment time was performed. Similar bacterial profiles and diversity were found in all samples regardless of the time point of the study. In this context, PCoA plots of bacterial profiles (at the ASV level) based on the Bray–Curtis distance matrix and the binary Jaccard distance matrix similarity analysis of the samples collected from both groups at different time points indicated that the variable time did not affect the clustering of the bacterial profiles (*p* = 0.999 and *p* = 1.00, respectively) (Figure S3, Supplementary Materials). This similar clustering, depending on the trial time, can also be visualized in the heatmap showing the relative abundance of the most abundant bacterial genera (x axis) detected (Figure S4, Supplementary Materials).

## 4. Discussion

The current study aimed at producing an innovative beverage from sprouted oat flour by fermentation with *L. plantarum* WCFS1. This strain was selected based on their versatile metabolism and capacity of growing on different vegetable sources together with its immunomodulatory and potential probiotic potentials [21]. The positive impact of germination and lactic acid fermentation on nutritional value, bioavailability of nutrients, and bioactive profile of grains has been extensively documented [15,22]. The combination of both processing technologies represents a novel strategy for producing cereal-based GF beverages.

The in vitro immunoblotting results indicate that toxic epitopes present in gluten-containing cereals that are involved in CD development are not present in the oat variety Meeri used in this study, since no reaction against AGA was observed in raw/sprouted oat flours and SOFB. These results suggest the potential lack of toxicity of SOFB for CD people. Several in vitro studies have established that few oat varieties are potentially harmful for CD people since they exert immunostimulatory activity on celiac T lymphocytes from peripheral blood and reacts to AGA. Both immunogenicity and immunoreactivity seemed to be cultivar-dependent [14,23]. The variation of prolamin genes and amino acid sequences observed in different oat cultivars likely result in differences in the degree of avenins immunogenicity [24]. Despite the existence of some potentially toxic oat cultivars for CD people, previous studies have concluded that, in general, the toxicity of the oat species is low [23,25]. The safety of SOFB for CD population was further confirmed by the human intervention trial which demonstrated that SOFB consumption did not induce the production of IgA anti-tTG antibodies in CD adult participants. These findings are consistent with earlier results from clinical trials performed in children and adults with CD which showed that consumption of gluten-uncontaminated oat products did not cause a deleterious serological antibody response [11,12,13]. The results obtained indicated that small-bowel mucosa morphology was not modified in the SOFB group during the intervention period, evidencing that the SOFB beverage was well tolerated by CD adults. In addition, no differences in IEL subset percentages were observed between the control and SOFB groups in histological samples from both second duodenal portion and duodenal bulb and the values were in accordance with those previously reported in small bowel mucosa of CD subjects on GF diets [18]. These results revealed the lack of toxicity of long-term SOFB intake in CD subjects, confirming earlier evidences reporting the absence of mucosal damage in CD individuals as a consequence of short- and long-term oat consumption [10,13]. 

Oxidative stress plays a key role in CD pathogenesis since gluten induces certain signaling transduction pathways in enterocyte that increase ROS levels, resulting in intracellular oxidative imbalance and synthesis of pro-inflammatory mediators [26]. Results of the present study indicated that neither SOFB nor the other oat beverages exhibited a cellular antioxidant effect in contrast with previous studies, demonstrating the cellular antioxidant activity of wholegrain and sprouted oat extracts in HepG2 cells exposed to *t*-BOOH and AAPH, mainly due to oat phenolic compounds [27,28]. Avenanthramides and ferulic acid are the most abundant phenolic compounds in oat grains and sprouts that exert their antioxidant properties directly scavenging/neutralizing oxygen radicals and indirectly activating the cellular antioxidant response triggering nuclear translocation of the transcription factor Nrf2 [29,30]. Despite earlier studies which have demonstrated that soluble phenolic concentration and antioxidant activity are improved after grain germination [15] and fermentation [22], the loss of antioxidant compounds throughout the production steps to obtain oat beverages might explain our results. The common steps in the production of plant-based milk substitutes are wet milling, filtration, the addition of ingredients, sterilization, homogenization, aseptic packaging, and cold storage [22]. In the present study, ROB, SOB, and SOFB production included the pasteurization of the oat slurry at 90 °C for 30 min. This thermal treatment could result in the degradation of phenolic compounds based on previous reports, which showed that thermal treatments (steaming and drying) often lead to a reduction in the content of phenolic acids and avenanthramides [22]. Additionally, pasteurization of oat slurries commonly applied in the preparation of plant-based beverages causes starch gelatinization and the increase in the slurry viscosity [31] which could be associated to a lower phenolic recovery during oat beverage production. Although the raw material includes both hydrophobic and hydrophilic phenolic compounds, another reason for the decrease in total polyphenols observed in most of the plant-based milk products is the low amounts of hydrophilic compounds [32]. There are few studies in the literature on the antioxidant capacity and phenolic compounds of nut and cereal milk substitutes. Total phenolic compounds of hazelnut and sesame decrease by about 42% and 82%, respectively, when the milk substitutes are produced [32,33]. 

Several shreds of evidence have demonstrated that gluten-dependent inflammation in CD duodenal mucosa results from a complex interaction of both innate and adaptive immunity [14]. In view of the importance of macrophages in innate immunity, the potential anti-inflammatory effects of SOFB and the other beverages developed was evaluated in a macrophage proinflammatory model. Gastrointestinal digests of all beverages were able to inhibit production of TNF-α, and ROB also reduced IL-6 release, suggesting their potential to revert the LPS-induced inflammatory response in macrophages. Similarly, other studies that have investigated the potential effect of oat-based foods or purified phytochemicals reported consistent anti-inflammatory effects that are commonly associated to β-glucans and phenolic compounds [30,34]. Oat anti-inflammatory compounds are inhibitors of NF-Κβ activation, suppressing the inflammatory response, oxidative and nitrosative pathways, and production of proinflammatory cytokines, including IL-1β, IL-6, and TNF-α [35]. IL-6 and TNF-α are inducers of tTG enzyme with a central role in the onset of CD as it is involved in the deamidation of gliadin peptides and the formation of immunotoxic peptides [3]. Therefore, it was addressed in the dietary intervention trial whether the observed anti-cellular inflammatory action of SOFB results in health benefits for CD people.

The intervention trial showed that SOFB consumption did not lead to body weight changes, consistent with a previous study not evidencing any weight-loss effect after oat intake [36]. Conversely, a weight-reducing effect after oat consumption for 2–12 months has been reported in type-2 diabetic and healthy individuals [37], likely due to its high β-glucan content, which may increase the viscosity of meals, reduce starch digestion, and decrease food intake by increasing satiety [38]. Similarly, our results reveal that serum levels of glucose, iron, ferritin, and vitamin B_12_ did not change in CD individuals as a consequence of SOFB consumption, while concentration of folic acid was significantly lower in the SOFB group than in the control one at the end of the study. It should be noted that even lower serum folic acid concentration was observed in SOFB participants compared with the control group at the completion of the study, and that the levels within the SOFB group did not suffer changes from 0 to 6 months. In agreement with our results, it has been reported that consumption of large oat amounts (350 g/week-100 g/day for 3–6 months) did not modify the levels of iron and ferritin in CD subjects on GF diet [13,39]. However, Kemppainen et al. [39] observed a significant decrease in serum vitamin B_12_ levels and an increase in erythrocyte folate in CD patients after oat consumption (100 g/day) for 6 months. These authors attributed the changes on the levels of these vitamins to modifications in the ingestion of vegetables during the intervention rather than to the effects of oat intake. Data regarding the effect of oat consumption on triglycerides and glucose levels are contradictory since some studies have reported a reduction in these parameters [39], but other investigations did not found any oat intake-derived effect [40].

Interestingly, the SOFB group exhibited lower serum cholesterol levels than the control group from the second month to the end of the intervention. Epidemiological and interventional studies clearly revealed the positive effect of oat consumption on the reduction in serum cholesterol levels. Oat cholesterol-lowering effects have been primarily ascribed to β-glucan due to its abilities to reduce intestinal cholesterol absorption, inhibit intestinal bile acids reabsorption, increase the synthesis of bile acids from cholesterol, and thus reduce circulating cholesterol levels and/or to modify intestinal microbiota composition resulting in an increased production of butyrate that may downregulate hepatic cholesterol synthesis [40]. The presence of β-glucan in SOFB, together with other oat components (proteins, sterols, and lipids) that can interact with cholesterol and/or bile acids [41], are likely responsible for the reduction in cholesterol levels in the SOFB group. The presence of *L. plantarum* WCFS1 in SOFB might also contribute to the reduction in cholesterol levels in CD patients, since the hypocholesterolemic effect of different *L. plantarum* strains, including WCFS1, has been previously reported [21]. The positive influence of SOFB on cholesterol levels suggests that its consumption might be useful in managing cholesterol levels that are often elevated in CD patients, but further studies in a higher celiac population need to be performed in order to confirm this hypothesis. However, it should be highlighted that the purpose of this study was to incorporate the SOFB to the diet without modifying the dietary habits of celiac participants, and therefore, the caloric and macronutrient intakes were not controlled during the intervention study. Although insignificant changes in dietary habits of celiac participants were observed in food records, the contribution of other dietary components on cholesterol reduction observed during the intervention study cannot be discarded.

It has been well established that dysbiosis, an imbalance of protective and pathogenic microorganisms in the host, is a risk factor of CD development. In this scenario, the increased abundance of *Bacteroides*, *Clostridium* spp., *Prevotella* spp., *Actinomyces* spp., and *E. coli*, and the decreased population of *Bifidobacterium* spp., *Lactobacillus* spp., and *Ruminococcus* are the often hallmarks of CD patients microbiota and the microbial imbalance persists despite adherence to the GF diet [42]. The results of the present work revealed that SOFB consumption significantly (*p* ≤ 0.05) increased the *Ruminococcus*, *Lactobacillus*, and *Subdoligranulum* populations in the CD individuals included in this study. Lactobacilli decreased the enteric pH by synthesizing lactic acid, which is required by butyrate-producing bacteria, such as those of the genera *Ruminococcus* and *Subdoligranulum*. In turn, butyrate-producing bacteria have the ability to promote the growth of lactobacilli [43]. The positive effect of SOFB on gut lactobacilli populations are in agreement with a previous study that reported the disappearance of intestinal dysbiosis characterized by lower lactobacilli populations on CD individuals after pure oat consumption [44]. The presence of β-glucan in SOFB [16] might be partially responsible for the lactobacilli enrichment due to its prebiotic properties. Resistant starch present in whole grain oats could also explain the increase in lactobacilli in celiac participants from the SOFB group due to its prebiotic activity. The high levels of probiotic *L. plantarum* WCFS1 in SOFB (8.9 log CFU/mL) [16] may have contributed to the increased load of lactobacilli in the gut of SOFB patients. Supplementation with probiotic lactobacilli strains have demonstrated a protective role against CD development, since they release peptidases that break down gluten peptides that cause a negative effect on the barrier function of intestinal cells and inflammation. Moreover, lactobacilli inhibit the production of pro-inflammatory cytokines, and reduce intestinal permeability to gluten proteins [45]. The increase in *Ruminococcus* populations in the SOFB group is of particular interest since this genus comprises keystone species that produce short-chain fatty acids (SCFA) that are essential for fiber and resistant starch degradation in the intestine, providing nutrients for the growth of butyrate-producing bacteria [46]. The enhanced *Subdoligranulum* populations, a genus capable of producing butyrate, in the SOFB group should also be highlighted. Butyrate is a major source of energy to the enterocytes and acts as a regulator of gene expression, immunomodulation, inflammation, differentiation in host cells, and exerts health effects on colonic epithelia [43]. Our findings demonstrated that SOFB combining oat prebiotic compounds and a probiotic lactic acid strain represent a promising approach for modulating intestinal microbiota and reduce proinflammatory state, thus improving the well-being and quality of life of CD people.

Limitations of this in vivo study include the low number and the high heterogeneity of CD adult participants. A higher sample size would be necessary to extrapolate the results obtained to all CD subjects. However, this is a preliminary pilot study that opens new opportunities to develop functional foods from oat suitable for celiac population, diversifying GF foods range and boosting the use of oat as GF ingredient. Further studies in a larger CD population and including a higher number of biomarkers of health status are necessary to demonstrate the potential application of SOFB and other symbiotic cereal beverages as adjunct in CD treatment.

## 5. Conclusions

The present investigation shows, for the first time, the in vitro and in vivo safety and health-promoting properties of a novel GF fermented beverage obtained from sprouted oat (SOFB) for CD individuals. SOFB did not exhibit immunoreactivity against AGA nor did it induce the production of IgA anti-tTG antibodies, intraephitelial lymphocytosis, and small-bowel mucosa damage in CD adults that consumed the beverage for 6 months. Although non-cellular antioxidant activity was observed, SOFB exhibited anti-inflammatory activity in a macrophage inflammation model. The intervention study performed in CD adults revealed that consumption of SOFB for 6 months did not influence serum biomarkers of nutritional/health status with the exception of serum cholesterol levels that were significantly lower in the SOFB group from the second month to the end of the study and folic and triglycerides levels that were lower at 6 months and at 0 and 6 months, respectively, in SOFB participants compared to the control group. Furthermore, the SOFB group showed positive changes in gut microbiota compared to the control one, including a particularly interesting increase in *Subdoligranulum* spp., *Ruminococcus* spp., and *Lactobacillus* spp. populations. These findings support the beneficial effects derived from SOFB consumption in CD individuals. Moreover, it should be emphasized that other dietary components might also contribute to the nutritional/health effects observed in celiac participants.

## Figures and Tables

**Table 1:** Demographic baseline characteristics of study participants.

Baseline Characteristics	Control Group (*n* = 5)	SOFB Group (*n* = 5)	*p*
Gender (male/female)	2/3	2/3	
Age (year)	45.20 ± 12.32	31.80 ± 12.52	0.126
Duration GF diet (year)	8.30 ± 9.36	8.80 ± 6.69	0.530
Weight (Kg)	72.20 ± 14.81	62.80 ± 5.80	0.168
Height (m)	1.68 ± 0.08	1.68 ± 0.08	0.727
Body mass index (Kg/m^2^)	25.54 ± 4.00	21.50 ± 1.99	0.078

Values are given as means ± SD. *p* ≤ 0.05 indicates significant differences between groups. GF: gluten-free.

**Table 2:** Effect of SOFB consumption on total serum IgA and IgA-class anti-tissue transglutaminase (IgA-tTG) antibodies in CD patients.

IgA Levels	Control Group	SOFB Group	*p*
Total serum IgA (mg/dL)			
0 months	200.00 ± 67.99 (146.0–288.0) ^a^	197.20 ± 60.26 (104.0–271.0) ^a^	1.000
2 months	188.40 ± 70.18 (126.0–286.0) ^a^	192.34 ± 61.95 (99.7–270.0) ^a^	1.000
4 months	196.00 ± 67.66 (143.0–288.0) ^a^	200.80 ± 66.38 (104.0–290.0) ^a^	0.841
6 months	202.20 ± 79.90 (1.26–311.0) ^a^	201.80 ± 67.15 (102.0–290.0) ^a^	1.000
IgA-tTG (U/mL)			
0 months	1.76 ± 0.62 (0.7–2.4) ^a^	2.72 ± 3.29 (0.5–8.5) ^a^	0.841
2 months	1.38 ± 0.62 (0.5–2.0) ^a^	2.64 ± 3.03 (0.9–8.0) ^a^	1.000
4 months	1.83 ± 0.54 (1.0–2.4) ^a^	2.39 ± 2.41 (1.0–6.7) ^a^	0.548
6 months	1.22 ± 0.56 (0.5–1.8) ^a^	2.55 ± 2.74 (0.2–7.2) ^a^	0.421

Values are given as means ± SD. Data in parentheses indicates minimum and maximum values for each variable. *p* ≤ 0.05 indicates significant differences between groups. ^a^ Similar superscript letters indicate not significant differences in the counts of each type of intraepithelial lymphocytes (IEL) between 0 and 6 months.

**Table 3:** Effect of SOFB consumption on counts of total intraepithelial lymphocytes (% relative to total epithelial cells), TCR γδ+ IEL (% relative to total IEL) and CD3^−^CD103^+^ IEL (% relative to total IEL).

	IEL Levels	Months	Control Group	SOFB Group	*p*
Second duodenal portion	Total IEL	0	10.80 ± 2.83 (6.80–12.90) ^a^	19.90 ± 9.84 (5.20–25.80) ^a^	0.343
		6	8.70 ± 4.04 (3.90–12.10) ^a^	8.24 ± 6.37 (2.50–0.40) ^a^	0.841
	TCR γδ+ IEL	0	28.28 ± 12.41 (19.40–46.50) ^a^	24.32 ± 9.55 (17.00–40.90) ^a^	0.413
		6	22.98 ± 6.77 (16.30–31.60) ^a^	29.16 ± 12.20 (16.50–46.70) ^a^	0.548
	CD3^−^CD103+ IEL	0	6.10 ± 5.74 (0.30–13.20) ^a^	3.12 ± 3.41 (0.40–8.40) ^a^	0.730
		6	7.44 ± 9.09 (1.20–23.50) ^a^	3.20 ± 4.15 (1.10–10.60) ^a^	0.222
Duodenal bulb	Total IEL	0	8.30 ± 4.51 (4.20–14.40) ^a^	11.57 ± 7.75 (5.9–20.40) ^a^	0.533
		6	5.83 ± 1.28 (4.40–7.40) ^a^	3.97 ± 2.35 (1.40–6.00) ^a^	0.800
	TCR γδ+ IEL	0	19.00 ± 15.67 (6.90–40.90) ^a^	24.46 ± 12.12 (14.90–38.10) ^a^	0.630
		6	20.80 ± 17.81 (8.20–47.20) ^a^	24.70 ± 10.07 (14.30–34.40) ^a^	0.533
	CD3^−^CD103^+^ IEL	0	3.23 ± 1.61 (1.80–4.90) ^a^	3.30 ± 4.33 (0.70–8.30) ^a^	0.629
		6	1.98 ± 0.86 (1.20–3.20) ^a^	1.96 ± 0.83 (1.30–2.90) ^a^	0.857

Values are given as means ± SD. Data in parentheses indicates minimum and maximum values for each variable. *p* ≤ 0.05 indicates significant differences between groups. ^a^ Similar superscript letters indicate not significant differences between time points. TCR γδ+ IEL: intraepithelial lymphocytes expressing the γδ T cell receptor; CD3^−^CD103 IEL: intraepithelial lymphocytes expressing CD103 molecule but not CD3 molecule.

**Table 4:** Effect of SOFB consumption on body weight and serum levels of glucose, iron, ferritin, vitamin B_12_, folic acid, hemoglobin, triglycerides, and total cholesterol in CD patients.

IgA Levels	Control Group	SOFB Group	*p*
Body weight (Kg)			
0 months	72.2 ± 14.8 (54.0–95.0) ^a^	62.8 ± 5.8 (54.0–68.0) ^a^	0.151
2 months	73.5 ± 16.8 (54.5–100.0) ^a^	62.7 ± 6.1 (52.5–68.0) ^a^	0.151
4 months	73.5 ± 16.4 (54.0–99.0) ^a^	63.1 ± 6.4 (52.5–69.0) ^a^	0.095
6 months	73.5 ± 16.7 (52.5–99.0) ^a^	62.8 ± 7.2 (51.0–70.0) ^a^	0.151
Glucose (μg/dL)			
0 months	89.4 ± 7.4 (79.0–97.0) ^a^	80.4 ± 6.9 (72.0–87.0) ^a^	0.151
2 months	83.8 ± 6.5 (73.0–90.0) ^a^	76.6 ± 10.7 (64.0–92.0) ^a^	0.222
4 months	88.2 ± 12.1 (75.0–106.0) ^a^	83.2 ± 1.7 (82.0–86.0) ^a^	0.690
6 months	84.2 ± 12.0 (71.0–95.0) ^a^	80.8 ± 5.9 (71.0–85.0) ^a^	0.421
Iron (mg/dL)			
0 months	98.6 ± 22.1 (79.0–132.0) ^a^	100.0 ± 26.5 (73.0–127.0) ^a^	0.841
2 months	86.2 ± 16.6 (64.0–108.0) ^a^	104.4 ± 33.8 (63.0–145.0) ^a^	0.420
4 months	88.0 ± 27.2 (53.0–119.0) ^a^	69.6 ± 43.8 (38.0–145.0) ^a^	0.222
6 months	101.6 ± 8.0 (95.0–114.0) ^a^	97.4 ± 61.7 (44.0–189.0) ^a^	0.690
Ferritin (ng/mL)			
0 months	62.9 ± 49.2 (20.9–143.5) ^a^	80.9 ± 66.5 (24.1–167.9) ^a^	0.917
2 months	73.6 ± 59.0 (17.3–164.9) ^a^	72.0 ± 60.1 (18.4–154.3) ^a^	0.917
4 months	72.1 ± 81.2 (19.1–214.0) ^a^	76.5 ± 69.4 (15.6–177.7) ^a^	0.917
6 months	80.5 ± 96.7 (13.1–247.3) ^a^	76.3 ± 69.2 (17.3–177.7) ^a^	0.754
Vitamin B_12_ (pg/mL)			
0 months	416.2 ± 143.8 (261.0–610.0) ^a^	407.8 ± 120.1 (261.0–589.0) ^a^	0.690
2 months	428.2 ± 164.2 (246.0–644.0) ^a^	398.0 ± 136.8 (217.0–589.0) ^a^	1.000
4 months	449.0 ± 202.7 (167.0–610.0) ^a^	393.8 ± 123.3 (218.0–544.0) ^a^	0.548
6 months	387.6 ± 121.4 (261.0–527.0) ^a^	417.2 ± 186.7 (274.0–728.0) ^a^	0.841
Folic acid (ng/mL)			
0 months	7.5 ± 3.7 (3.7–13.2) ^a^	5.1 ± 2.6 (2.8–9.4) ^a^	0.310
2 months	9.8 ± 4.2 (6.1–15.9) ^a^	5.7 ± 3.0 (2.1–9.0) ^a^	0.222
4 months	6.6 ± 1.9 (4.5–8.8) ^a^	4.9 ± 2.4 (2.1–8.7) ^a^	0.220
6 months	8.0 ± 2.2 (5.4–10.7) ^a^	5.1 ± 2.1 (3.1–8.7) ^a^	0.032
Hemoglobin (g/dL)			
0 months	14.4 ± 1.3 (12.5–16.2) ^a^	14.1 ± 0.8 (13.3–15.2) ^a^	0.690
2 months	14.4 ± 1.5 (12.8–16.7) ^a^	12.0 ± 4.3 (4.4–14.3) ^a^	0.310
4 months	14.3 ± 1.3 (12.8–16.0) ^a^	12.1 ± 4.1 (4.9–14.3) ^a^	0.548
6 months	14.2 ± 1.9 (11.9–16.4) ^a^	13.9 ± 0.4 (13.3–14.3) ^a^	0.841
Triglycerides (mg/mL)			
0 months	133.8 ± 43.6 (64.0–174.0) ^a^	53.6 ± 19.3 (34.0–82.0) ^a^	0.016
2 months	110.8 ± 47.8 (68.0–190.0) ^a^	69.6 ± 46.0 (33.0–150.0) ^a^	0.095
4 months	94.8 ± 26.9 (64.0–130.0) ^a^	81.0 ± 48.0 (30.0–150.0) ^a^	0.069
6 months	116.4 ± 59.7 (51.0–207.0) ^a^	51.6 ± 20.4 (41.0–88.0) ^a^	0.016
Total cholesterol (mg/dL)			
0 months	198.0 ± 41.2 (147.0–262.0) ^a^	147.2 ± 22.4 (119.0–170.0) ^a^	0.060
2 months	188.2 ± 25.9 (158.0–215.0) ^a^	132.0 ± 28.4 (94.0–163.0) ^a^	0.016
4 months	189.0 ± 42.3 (147.0–260.0) ^a^	137.4 ± 18.32 (115.0–158.0) ^a^	0.032
6 months	199.0 ± 30.2 (163.0–235.0) ^a^	146.6 ± 21.4 (124.0–175.0) ^a^	0.016

Values are given as means ± SD. Data in parentheses indicates minimum and maximum values for each variable. *p* ≤ 0.05 indicates significant differences between groups. ^a^ Similar superscript letters indicate non-significant differences between time points.

**Table 5:** Relative frequencies, medians and interquartile range (IQR) of the most abundant bacterial phyla (bold) and genera detected in fecal samples from the control and SOFB groups.

Phylum/Genus	Control Group		SOFB Group		*p*
	*n* (%)	Median (IQR)	*n* (%) ^#^	Median (IQR)	
***Firmicutes***	20 (100%)	88.78 (80.85–92.2)	20 (100%)	91.46 (89.03–93.52)	0.081
*Blautia*	20 (100%)	9.79 (7–12.58)	20 (100%)	10.07 (6.32–14.58)	0.95
*Subdoligranulum*	20 (100%)	2.72 (1.42–6.05)	20 (100%)	6.99 (4.72–8.93)	**0.002**
*Faecalibacterium*	16 (80%)	3.83 (0.64–7.9)	20 (100%)	3.85 (1.61–8.07)	0.6
*Agathobacter*	20 (100%)	2.04 (1.58–5.25)	20 (100%)	1.96 (1.18–7.05)	0.82
*Dorea*	20 (100%)	3.19 (2.56–5.56)	20 (100%)	3.87 (1.6–5.11)	0.56
*Anaerostipes*	20 (100%)	3.41 (1.19–8.27)	20 (100%)	1.34 (0.6–3.13)	**0.02**
*Streptococcus*	20 (100%)	1.9 (1.23–4.22)	20 (100%)	1.11 (0.54–3.44)	0.15
*Erysipelotrichaceae*	20 (100%)	4.02 (1.75–4.83)	19 (95%)	2.61 (0.66–4.04)	0.12
*Ruminococcus*	6 (30%)	<0.01 (<0.01–0.01)	17 (85%)	5.28 (0.16–7.17)	**<0.001**
*Lactobacillus*	15 (75%)	0.04 (<0.01–0.13)	17 (85%)	0.25 (0.05–3.11)	**0.028**
*Clostridium*	15 (75%)	0.4 (0.01–1.19)	20 (100%)	0.38 (0.18–2.08)	0.25
*Romboutsia*	17 (85%)	0.78 (0.38–1.48)	20 (100%)	1.02 (0.43–2.62)	0.39
*Holdemanella*	4 (20%)	<0.01 (<0.01–<0.01)	8 (40%)	<0.01 (<0.01–1.56)	0.43
*Monoglobus*	16 (80%)	1.29 (0.1–1.89)	19 (95%)	0.95 (0.3–2.32)	0.64
*Coprococcus*	17 (85%)	1.35 (0.98–1.8)	20 (100%)	1.8 (1.17–2.1)	0.13
*Catenibacterium*	5 (25%)	<0.01 (<0.01–<0.01)	4 (20%)	<0.01 (<0.01–<0.01)	0.81
***Actinobacteria***	20 (100%)	5.69 (4.41–6.87)	20 (100%)	5.43 (3.58–6.51)	0.24
*Bifidobacterium*	20 (100%)	1.96 (0.79–4.09)	19 (95%)	1.72 (0.52–3.16)	0.58
***Bacterioidota***	20 (100%)	3.01 (0.86–11.09)	19 (95%)	2.17 (0.58–4.13	0.3
*Bacteroides*	18 (90%)	2.5 (0.61–7.67)	18 (90%)	1.24 (0.25–2.58)	0.16
***Proteobacteria***	18 (90%)	0.17 (0.05–0.67)	20 (100%)	0.25 (0.14–0.64)	36
***Fusobacteriota***	10 (50%)	0.01 (<0.01–0.11)	3 (15%)	<0.01 (<0.01–<0.01)	**0.011**
Minor phyla	19 (95%)	0.07 (0.02–0.21)	16 (80%)	0.06 (0.01–0.6)	0.86
Minor genera	20 (100%)	15.44 (14.57–21.34)	20 (100%)	17.55 (13.63–21.97)	0.97
Unclassified genera	20 (100%)	17.55 (13.63–21.97)	20 (100%)	13.3 (10.94–14.75)	**0.001**

^#^ (%): number of samples in which the phylum/genus was detected (relative frequency of detection). *p* ≤ 0.05 indicates significant differences between groups (indicated in bold).

## Data Availability

The data supporting results are confidential.

## Supplementary Materials

The following are available online at https://www.mdpi.com/article/10.3390/nu13082522/s1, Figure S1: In vitro and in vivo evaluation of safety and nutritional/health benefits of SOFB, Figure S2: Feces microbial diversity as assessed using the Shannon (a) and Simpson (b) diversity indices comparing control (blue boxes) and SOFB (yellow boxes) groups, Figure S3: Comparison, at the ASVs level, of the beta diversity for samples collected from control (a) and SOFB (b) groups at different times. Principal coordinate analysis (PCoA) plots of bacterial profiles based on the Bray-Curtis similarity analysis (relative abundance). The values on each axis label in graphs represent the percentage of the total variance explained by that axis, Figure S4: Heatmap plot representing the hierarchical clustering, at the genus level, of the feces samples from the SOFB group collected at different times.

## References

[1] Catassi C., Fasano A. (2012). Clincal Practice. Celiac disease. N. Engl. J. Med..

[2] Dotsenko V., Oittinen M., Taavela J., Popp A., Peräaho M., Staff S., Sarin J., Leon F., Isola J., Mäki M. (2021). Genome-wide transcriptomic analysis of intestinal mucosa in celiac disese patients on a gluten-free diet and postgluten challenge. Cell. Mol. Gastroenterol. Hepatol..

[3] Kramer K., Yeboah-Awudzi M., Magazine N., King J.M., Xu Z., Losso J.N. (2019). Procyanidin B2 rich cocoa extracts inhibit inflammation in Caco-2 cell model of in vitro celiac disease by down-regulating interferon-gamma- or gliadin peptide 31-43-induced transglutaminase-2 and interleukin-15. J. Func. Foods.

[4] Caio G., Volta U., Sapone A., Leffler D., Giorgio R., Catassi C., Fasano A. (2019). Celiac disease: A comprehensive current review. BMC Med..

[5] Elliot C. (2018). The nutritional quality of gluten-free products for children. Pediatrics.

[6] Lionetti E., Antonucci N., Marinelli M., Bartolomei B., Franceschini E., Gatti S., Naspi Catassi G., Verma A.K., Monachesi C., Catassi C. (2019). Nutritional status, dietary intake, and adherence to the Mediterranean diet on children with celiac disease on a gluten-free diet: A case-control prospective study. Nutrients.

[7] Larretxi I., Simon E., Benjumea L., Miranda J., Bustamante M.A., Lasa A., Euzaguirre F.J., Churruca I. (2019). Gluten-free-rendered products contribute to imbalanced diets in children and adolescents with celiac disease. Eur. J. Nutr..

[8] Arentz-Hansen H., Fleckenstein B., Molberg O., Scott H., Koning F., Jung G., Roepstorff P., Lundin K.E., Sollid L.M. (2004). The molecular basis for oat intolerance in patients with celiac disease. PLoS Med..

[9] Lundin K.E.A., Nilsen E.M., Scott H.G., Løberg E.M., Gjøen A., Bratlie J., Skar V., Mendez E., Løvik A., Kett K. (2003). Oats induced villous atrophy in coeliac disease. Gut.

[10] Kaukinen K., Collin P., Huhtala H., Mäki M. (2013). Long-term consumption of oats in adult celiac disease patients. Nutrients.

[11] Koskinen O., Villanen M., Korponay-Szabo I., Lindfords K., Mäki M., Kaukinen K. (2009). Oats do no induce systemic or mucosa autoantibody response in children with coeliac disease. J. Pediatr. Gastroenterol. Nutr..

[12] Lionetti E., Gatti S., Galeazzi T., Caporelli N., Francavilla R., Cucchiara S., Roggero P., Malamisura B., Iacono G., Tomarchiio S. (2018). Safety of oats in children with celac disease: A double-blind, randomized, placebo-controlled trial. J. Pediatr..

[13] Sey M.S.L., Parfitt J., Gregor J. (2011). Prospective study of clinical and histological safety of pure and uncontaminated Canadian oats in the management of celiac disease. J. Parenter. Enteral Nutr..

[14] Silano M., Peñas E., Uberti F., Manferdelli S., del Pinto T., Felli C., Budelli A., Vincentini O., Restani P. (2014). Diversity of oat varieties in eliciting early inflammatory events in celiac disease. Eur. J. Nutr..

[15] Aparicio-García N., Martínez-Villaluenga C., Frias J., Peñas E. (2021). Sprouted oat as a potential gluten-free ingredient with enhanced nutritional and bioactive properties. Food Chem..

[16] Aparicio-García N., Martínez-Villaluenga C., Frias J., Peñas E. (2021). Production and characterization of a novel gluten-free fermented beverage based on sprouted oat flour. Foods.

[17] Brodkorb A., Egger L., Alminger M., Alvito P., Assunção R., Ballance S., Bohn T., Bourlieu-Lacanal C., Boutrou R., Carrière F. (2019). INFOGEST static in vitro simulation of gastrointestinal food digestion. Nat. Protoc..

[18] De Andrés A., Camarero C., Roy G. (2015). Distal duodendenum versus duodenal bulb: Intraepihtelial lymphocytes have something to say in celiac disease diagnosis. Dig. Dis. Sci..

[19] Castro I., Alba C., Fernández L., García A.J., Rodríguez J.M. (2020). Culture-dependent and metataxonomic analysis of milk rom red deer (*Cervus elaphus*). Int. Dairy J..

[20] Klindworth A., Pruesse E., Schweer T., Peplies J., Quast C., Horn M., Glöckner F.O. (2013). Evaluation of general 16S ribosomal RNA gene PCR primers for classical and next-generation sequencing-based diversity studies. Nucleic Acids Res..

[21] Van den Nieuwboer M., van Hemert S., Claassen E., de Vos W.M. (2016). *Lactobacillus plantarum* WCFS1 and its host interaction: A dozen years after the genome. Microbial. Biotechnol..

[22] Aydar E.F., Tutuncu S., Ozcelik B. (2020). Plant-based milk substitutes: Bioactive compounds, conventional and novel processes, bioavailability studies, and health effects. J. Func. Foods.

[23] Ballabio C., Uberti F., Manferdelli S., Vacca E., Boggini G., Radaelli R., Catassi C., Lionetti E., Peñas E., Restani P. (2011). Molecular characterization of 36 oat varieties and in vitro assessment of their suitability for celiac diet. J. Cereal Sci..

[24] Real A., Comino I., Lorenzo L., Merchán F., Gil-Humanes J., Giménez M.J., López-Casado M.A., Torres M.I., Cebolla A., Sousa C. (2012). Molecular and immunological characerization of gluten proteins isolated from oat cultivars that differ in toxicity for celiac disease. PLoS ONE.

[25] Giménez M.J., Real A., García-Molina M.D., Sousa C., Barro F. (2017). Characterization of celiac disease related oat proteins: Bases for the development of high quality oat varieties suitable for celiac patients. Sci. Rep..

[26] Luciani A., Villella V.R., Vasaturo A., Giardino I., Pettoello-Mantovani M., Guido S., Cexus O.N., Peake N., Londei M., Quaratino S. (2010). Lysosomal accumulation of gliadin p31–43 peptide induces oxidative stress and tissue transglutaminase-mediated PPARgamma downregulation intestinal epithelial cells and coeliac mucosa. Gut.

[27] Chen C., Wang L., Wang R., Luo X., Li Y., Li J., Li Y., Chen Z. (2018). Phenolic contents, cellular antioxidant activity and antiproliferative capacity of different varieties of oats. Food Chem..

[28] Lee J.H., Lee B.-K., Park H.-H., Lee B.W., Woo K.S., Kim H.-J., Han S.-I., Lee Y.Y. (2019). Oat germination and ultrafiltration process improves the polyphenol and avenanthramide contents with protective effect in oxidative-damaged HepG2 cells. J. Food Biochem..

[29] Song Y., Wen L., Sun J., Bai W., Jiao R., Hu Y., Peng X., He Y., Ou S. (2016). Cytoprotective mechanism of ferulic acid against high glucose-induced oxidative stress in cardiomyocytes and hepatocytes. Food Nutr. Res..

[30] Wang C., Eskiw C.H. (2019). Cytoprotective effects of Avenathramide C against oxidative and inflammatory stress in normal human dermal fibroblasts. Sci. Rep..

[31] Deswal A., Deora N.S., Mishra H.N. (2014). Effect of concentration and temperature on the rheological properties of oat milk. Food Bioproc. Tech..

[32] Fitrotin U., Utami T., Hastuti P., Santoso U. (2015). Antioxidant properties of fermented sesame milk using *Lactobacillus plantarum* Dad 13. Int. Res. J. Biol. Sci..

[33] Alasalvar C., Bolling B.W. (2015). Review of nut phytochemicals, fat-soluble bioactives, antioxidant components and health effects. Br. J. Nut..

[34] Suchecka D., Błaszczyk K., Harasym J., Gudej S., Wilczak J., Gromadzka-Ostrowska J. (2017). Impact of purified oat 1-3,1-4-β-d-glucan of different molecular weight on alleviation of inflammation parameters during gastritis. J. Func. Foods..

[35] Chu Y.-F., Wise M.L., Gulvady A.A., Chang T., Kendra D.F., Jan-Willem van Klinken B., Shi Y., O’Shea M. (2013). In vitro antioxidant capacity and anti-inflammatory activity of seven common oats. Food Chem..

[36] Charlton K.E., Tapsell L.C., Batterham M.J., O’Shea J., Thorne R., Beck E., Tosh S.M. (2012). Effect of 6 weeks’ consumption of β-glucan-rich oat products on cholesterol levels in mildly hypercholesterolaemic overweight adults. Brit. J. Nutr..

[37] Schuster J., Benincá G., Vitorazzi R., Morelo Dal Bosco S. (2015). Effects of oats on lipid profile, insulin resistance and weight loss. Nutr. Hosp..

[38] Geliebter A., Grillot C.L., Aviram-Friedman R., Haq S., Yahav E., Hashim S.A. (2015). Effects of oatmeal and corn flakes cereal breakfasts on satiety, gastric emptying, glucose, and appetite-related hormones. Ann. Nutr. Metab..

[39] Kemppainen T., Heikkinen M., Ristikankare M., Kosma V.-M., Julkunen R. (2009). Effect of unkilned and large amounts of oats on nutritional state of celiac patients in remission. e-Spen.

[40] Zhu X., Sun X., Wang M., Zhang C., Cao Y., Mo G., Liang J., Zhu S. (2015). Quantitative assessment of the effects of beta-glucan consumption on serum lipid profile and glucose level in hypercholesterolemic subjects. Nutr. Metab. Cardiovasc. Dis..

[41] Andersson K.E., Chawade A., Thuresson N., Rascon A., Öste R., Sterner O., Olsson O., Hellstrand P. (2017). Wholegrain oat diet changes the expression of genes associated with intestinal bile acid transport. Mol. Nutr. Food Res..

[42] Bodkhe R., Shetty S.A., Dhotre D.P., Verma A.K., Bhatia K., Mishra A., Kaur G., Pande P., Bangarusamy D.K., Santosh B.P. (2019). Comparison of small gut and whole gut microbiota of first-degree relatives with adult celiac disease patients and controls. Front. Microbiol..

[43] Pecora F., Persico F., Gismondi P., Fornaroli F., Luliano K.S., de’Angelis G.L., Esposito S.D. (2020). Gut microbiota in celiac disease: Is there anay role for probiotics?. Front. Immunol..

[44] Nylund L., Hakkola S., Lahti L., Salminen S., Kalliomäki M., Yang B., Linderborg K.M. (2020). Diet, perceived intestinal well-being and compositions of fecal microbiota and short chain fatty acids in oat-using subjects with celiac disease or gluten sensitivity. Nutrients.

[45] Giorgi A., Cerrone R., Capobianco D., Filardo S., Mancini P., Zanni F., Fanelli S., Mastromarino P., Mosca L. (2020). A probiotic preparation hydrolyzes gliadin and protects intestinal cells from the toxicity of pro-inflammatory peptides. Nutrients.

[46] Ze X., Duncan S.H., Louis P., Flint H.J. (2012). *Ruminococcus bromii* is a keystone species for the degradation of resistant starch in the human colon. ISME J..

