# Supplementary material for: A Novel Sprouted Oat Fermented Beverage: Evaluation of Safety and Health Benefits for Celiac Individuals
Source: Nutrients. 2021 Jul 23;13(8):2522. doi: 10.3390/nu13082522 (PMC8401588; doi:10.3390/nu13082522)

Figure S1. *In vitro* and *in vivo* evaluation of safety and nutritional/health benefits of SOFB

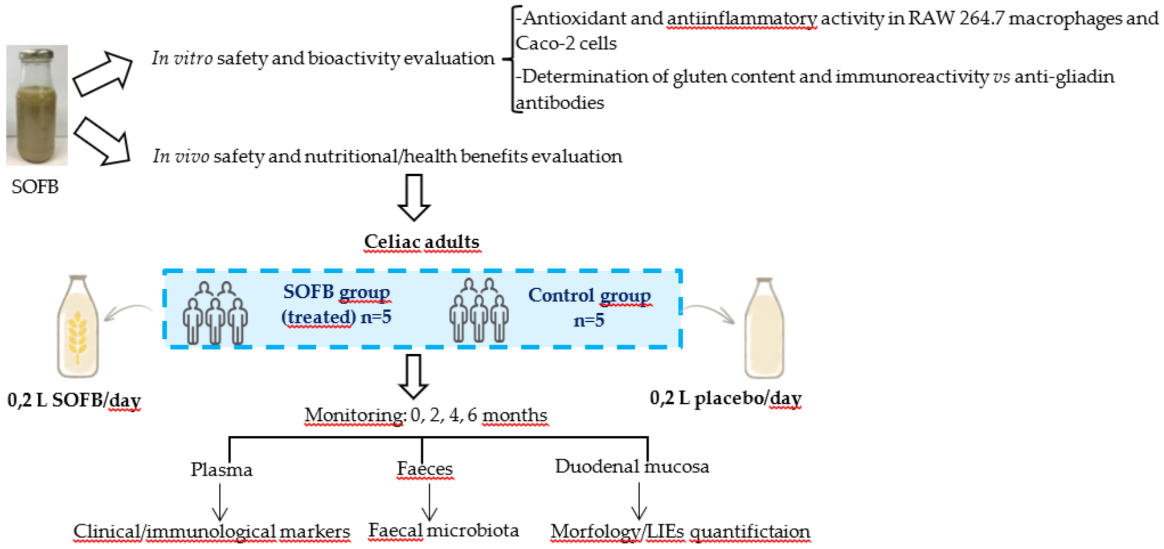

Figure S2. Feces microbial diversity as assessed using the Shannon (a) and Simpson (b) diversity indices comparing control (blue boxes) and SOFB (yellow boxes) groups

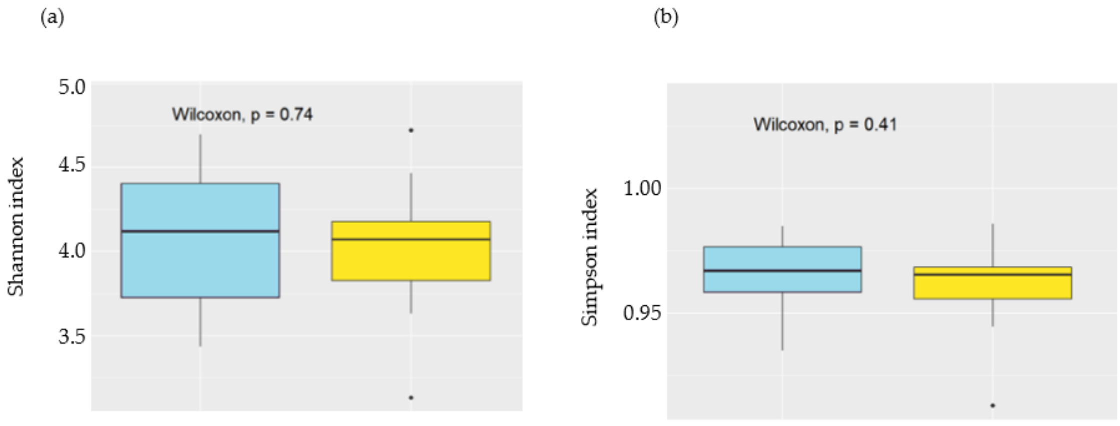

Figure S3. Comparison, at the ASVs level, of the beta diversity for samples collected from control (a) and SOFB (b) groups at different times. Principal coordinate analysis (PCoA) plots of bacterial profiles based on the Bray-Curtis similarity analysis (relative abundance). The values on each axis label in graphs represent the percentage of the total variance explained by that axis.

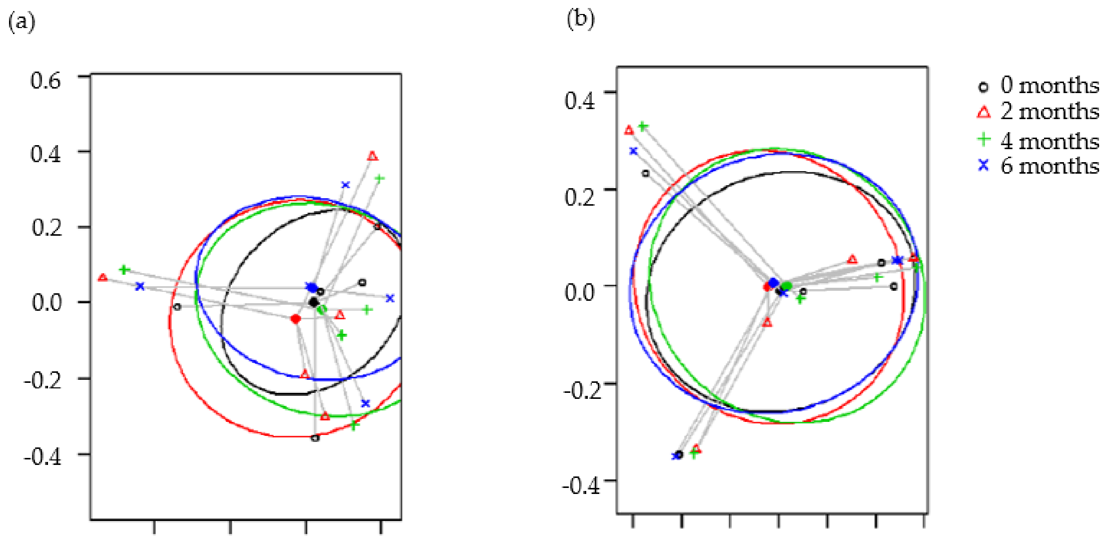

Figure S4. Heatmap plot representing the hierarchical clustering, at the genus level, of the feces samples from SOFB group collected at different times.

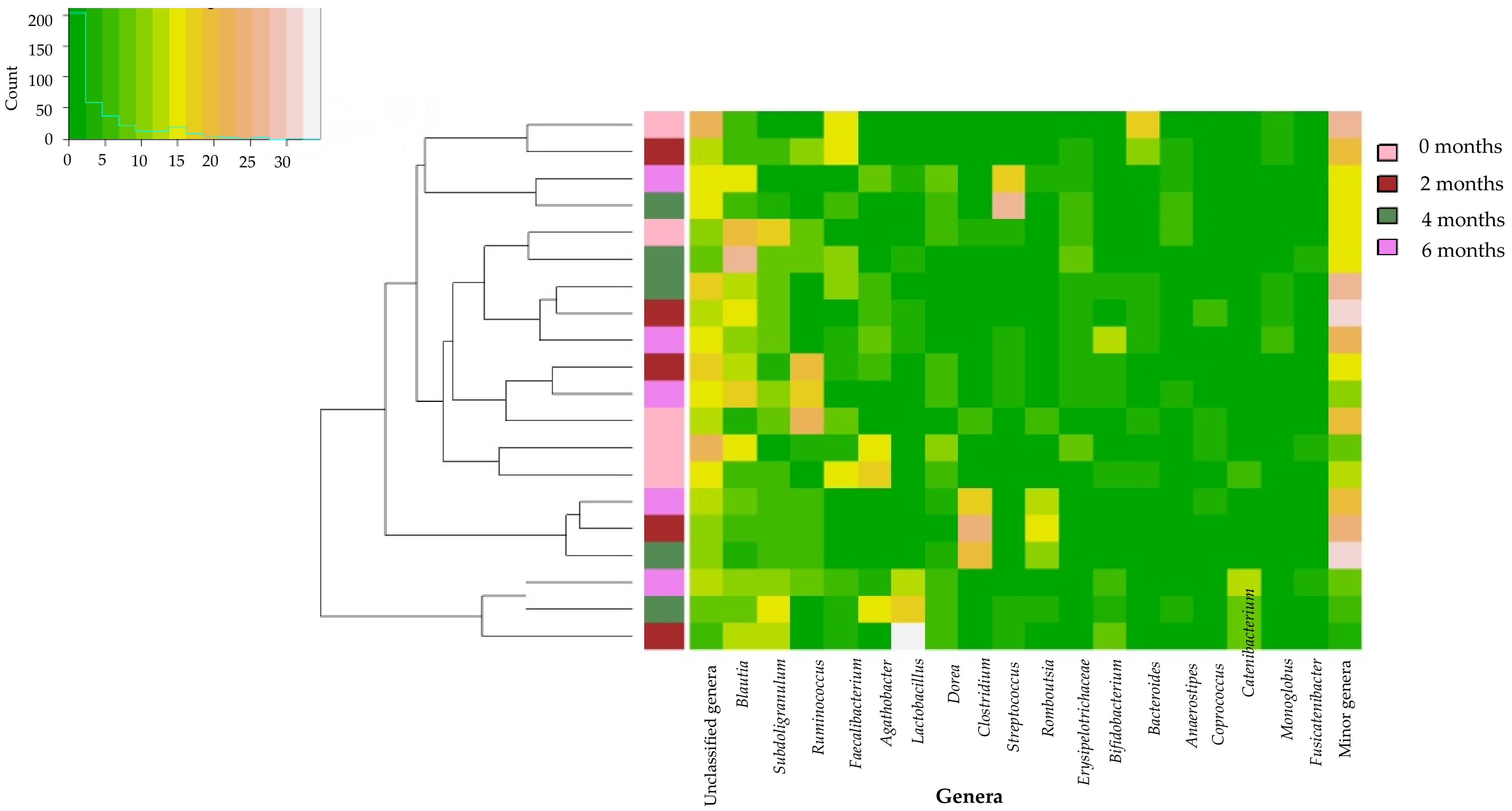

Supplement: Supplementary file 1 [file nutrients-13-02522-s001.zip › nutrients-1311884-supplementary.pdf]
